# Genetic variation and heritability of haploid frailty in maize

**DOI:** 10.3389/fpls.2025.1572901

**Published:** 2025-06-03

**Authors:** Recep Yavuz, Hao Wang, Mercy Fakude, Abil Dermail, Ursula Karoline Frei, Peng Liu, Thomas Lübberstedt

**Affiliations:** ^1^ Department of Agronomy, Iowa State University, Ames, IA, United States; ^2^ Department of Statistics, Iowa State University, Ames, IA, United States; ^3^ Department of Agronomy, Faculty of Agriculture, Khon Kaen University, Khon Kaen, Thailand

**Keywords:** doubled haploid, haploid frailty, haploid induction, heritability, plant height, spontaneous haploid genome doubling

## Abstract

This research investigated the variation in haploid frailty (%HF), which is the difference in trait performance between isogenic haploid and diploid maize lines, and the heritability of haploid frailty for different agronomic traits. A total of 48 isogenic pairs was evaluated in three environments, and 192 isogenic line pairs were evaluated in two environments for plant height (PH), ear height (EH), flag leaf length (FLL) and width (FLW), tassel length (TL), spike length (SL), stem diameter (SD) and tassel branch (TB) number. We found that the *qshgd1* locus, associated with spontaneous haploid genome doubling (SHGD), plays a crucial role in improving haploid performance by reducing %HF and promoting diploid-like vigor. The BS39+SHGD genotypes exhibited significantly lower HF% rates compared to the BS39 group, with consistent reductions across multiple traits. Environmental factors also contributed to %HF variation, but genetic influences such as the presence of SHGD proved to have a greater impact on haploid frailty. Leveraging SHGD to enhance both vigor and fertility of haploid plants, is likely to benefit breeding programs in maize and perhaps other crops by more economic and efficient production of DH lines.

## 1 Introduction

Breeding programs of important major crops use doubled haploid (DH) lines to speed up the breeding process (e.g., maize, wheat, barley, canola). Completely homozygous and homogeneous DH lines are obtained within only two generations. The two major limitations in working with haploid plants are their sterility and reduced vigor (Liu et al., 2016). We will call the weaker performance of haploids vs. isogenic diploids, “haploid frailty.” The extent of haploid frailty can be quantified for any trait as haploid versus isogenic diploid performance. One unexplored aspect in the process of developing DH lines is the genetic variability for haploid frailty.

Although the multicellular haploid generation went extinct in the life cycle of vascular plants roughly 400 million years ago (Kenrick and Crane, 1997; Niklas and Kutschera, 2010), it persists in the haploid-diploid life cycles (also known as biphasic life cycles) commonly found, for example, in many green, red and brown seaweeds (Bell, 1982). The most notable conclusion from studies of algae with extended both haploid and diploid life stages is, that haploids are not always inferior, but can retain significant presence and retain dominance in different species, depending on environmental conditions.

The study of haploids and the effects of ploidy has been a subject of interest in plant genetics for decades (Chase, 1964; Guo et al., 1996; Miller et al., 2012; Dermail et al., 2024). In maize, early observations by Randolph et al. (1944) highlighted a doubling in volume between diploid and tetraploid structures, a phenomenon referred to as the Gigas state in tetraploids. Despite their larger size, tetraploid structures were found to contain a similar number of parts and showed no deformities. Chase (1964) postulated that maize haploids might exhibit a proportional decrease in volume without deformities. However, his findings revealed that haploids were smaller than expected, approximately 11% below theoretical expectations, a discrepancy termed the odd-ploidy effect phenomenon (Guo et al., 1996).

Further investigations by Chase (1964) into isogenic haploid and diploid maize plants from six inbred lines showed reductions in plant parts, linear, area, and volume measures in haploids, with volume reduction also resulting in decreased organ weight. Reduced vigor in haploid plants has been reported not only in maize but also in *Arabidopsis thaliana* (Ravi and Chan, 2010) and across various species (Dunwell, 2010), suggesting that haploidy reduces higher plant vigor.

Haploid higher plants are not only less vigorous, but usually also sterile. However, spontaneous haploid genome doubling (SHGD) has been reported in maize (Kleiber et al., 2012; Ren et al., 2017, 2020; Sugihara et al., 2013; Wu et al., 2014, 2017; Yang et al., 2019; De la Fuente et al., 2020; Trampe et al., 2020), as well as in other grass species, and has likely been an important factor in the formation of some of our polyploid crops (Castillo et al., 2009). SHGD has been reported for tropical and elite temperate maize with European and North American origin (Kleiber et al., 2012), as well as Chinese germplasm (Ren et al., 2017; Wu et al., 2017). A higher level of haploid fertility compared to maize was reported for other species, such as *A. thaliana* (Ravi and Chan, 2010).

This research aims to better understand the limitations of haploids in maize, examine genetic variation for haploid frailty, and determine the heritability of haploid frailty for different agronomic traits. The specific objectives of this study were (i) to develop and evaluate a set of perfect isogenic haploid and diploid maize line pairs, (ii) to assess the heritability of haploid frailty for different agronomic traits in maize, and (iii) to determine the impact of a major quantitative trait locus (QTL) for spontaneous haploid genome doubling (*qshgd1*) on haploid frailty. Our study is aimed at selecting and developing more vigorous haploid maize lines with reduced haploid frailty in future, contributing to the development of more vigorous maize DH lines.

## 2 Materials and methods

### 2.1 Plant materials

The BS39 population was originally derived from five exotic Tusón accessions and was adapted to Midwestern photoperiod conditions through multiple generations of crossbreeding (Hallauer and Carena, 2016). To introduce the SHGD trait, BS39-SHGD-DH lines were developed through a cross between BS39 and pure inbred A427 at the Iowa State University-DH Facility. Inbred A427 (~78% high maternal frequency, HMF) served as the SHGD donor, mediated by a major quantitative trait locus on chromosome 5 (qshgd1) (Trampe et al., 2020; Foster et al., 2024). Both BS39-derived and BS39-SHGD-derived DH lines were genotyped using genotyping-by-sequencing (GBS) (Verzegnazzi et al., 2021; Santos et al., 2022).

In the summer of 2022, 48 isogenic lines (24 BS39 and 24 BS39-SHGD) with sufficient seed availability was selected for first year experiment. Additionally, during the summer of 2022, a total of 228 inbred lines were established, consisting of 85 lines derived solely from the BS39 population (BS39-inbreds) and 143 lines derived from the BS39 × A427 cross (BS39-SHGD-inbreds). These inbred lines were crossed with the inducer line BHI306 to generate haploid seeds. Manual haploid selection was performed based on the expression of R1-Navajo pigmentation in the embryo and aleurone.

Following haploid selection, haploid isogenic lines (HILs) were successfully obtained for 192 of the 228 inbred lines, with 66 derived from BS39 (BS39-HILs) and 126 from BS39-SHGD (BS39-SHGD-HILs). The selection process ensured that these 192 isogenic pairs maintained their respective genetic backgrounds, meaning they represent distinct genetic groups rather than overlapping subsets.

### 2.2 Experimental setup and data collection

In the summer of 2022, 48 HIL – DH line pairs were sown at the Agricultural Engineering and Agronomy Research Farm in Boone, IA. Measurements of plant height and ear height were recorded during the same growing season. During the summer of 2023, a larger group of 192 HIL – DH lines pairs was planted at two different timepoints (Rep1-05/18/2023; Rep2-05/29/2023) at the Agricultural Engineering and Agronomy Research Farm in Boone, IA. Those 192 isogenic line pairs included the 48 pairs evaluated in 2022. The staggered plantings were considered as two environments. Eight traits (PH, EH, FLL, FLW, TL, SL, SD, TB) were measured during the 2023 growing season.

Trait measurements were conducted for both diploid and haploid lines across key growth stages. PH was recorded at the VT and R1 stages by measuring from ground level to the tip of the tassel using a ruler. EH was measured during the VT and R1 stages from ground level to the base of the ear using a measuring tape. SD was measured at the R2 stage with a caliper, positioned around the second internode from bottom. FLL was measured at the R2 stage from the leaf collar to the tip using a tape measure, while FLW was determined at the widest point of the leaf at the same stages. TL was measured from the base to the tip of the tassel at the R2 stage using a measuring tape. SL was similarly recorded from the base to the tip of the spike at the R2 stage. TB were counted at the R1 stage by tallying the lateral branches emerging from the central spike (Fakude et al. (in preparation)).

Our study used a completely randomized design, where isogenic line pairs were randomized within each environment. Each line was planted with twenty seeds, with a spacing of 10 cm between seeds within each row and a spacing of 0.7 meters between rows.

Dataset 1 (2022 & 2023 Data) consists of PH and EH obtained from all three environments (2022 and the two staggered plantings in 2023) for 48 isogenic line pairs, each with three biological replications of haploid and diploid lines. Each environment represented a separate field block. Within each environment, biological replications were managed independently rather than being nested. Each genotype was evaluated with three biological replicates per environment, ensuring consistency in data collection across locations. In this dataset, the isogenic line pairs were evenly distributed between the two groups, with 50% representing BS39 and the other 50% representing BS39+SHGD. This dataset set includes 864 entries in total.

Dataset 2 (2023 Data) contains data from the two environments in 2023 and includes eight traits measured for the 192 isogenic line pairs, each with three biological replicates for haploid and DH lines in each environment (2304 entries). In this set, 66% of the isogenic line pairs belong to BS39, while the remaining 34% are BS39+SHGD.

The percentage of haploid frailty (%HF), reflects the reduced trait performance of haploid lines relative to the performance of isogenic diploid lines. We calculated the percentage of %HF using the following formula, taking PH as an example:


((1–(PHhaploid/PHdiploid))×100=%HF,

where PHhaploid and PHdiploid correspond to the average plant height across three biological replications for the haploid and its isogenic DH line, respectively. For each isogenic pair in each environment, we have one value of %HF calculated based on the average trait of the three plants for haploid and diploid.

### 2.3 Statistical analysis

We conducted linear mixed-effects model analysis to analyze %HF. Because the two groups (BS39 and BS39+SHGD) may demonstrate different characteristics related to haploid frailty, we conducted both joint and separate analyses with respect to the two groups.

#### 2.3.1 Linear mixed effects model analysis

For both Dataset 1 and Dataset 2, we fit the following linear mixed-effects model for %HF:


$$y_{ij}=\mu +g_{i}+r_{j}+{Є}_{ij},$$

where $y_{ij}$ is the haploid frailty (HF%) calculated for the *i*-th genotype from *j*-th environment, μ is the overall mean haploid frailty, $g_{i}∼N(0,\sigma_{g}^{2})$ is the random genotype effect, $r_{j}∼N(0,\sigma_{r}^{2})$ is the random environment effect, and ${Є}_{ij}∼N(0,\sigma_{e}^{2})$ is the random residual error. Because only one value of %HF was calculated for each isogenic pair in each environment, we cannot separately estimate the variance component corresponding to the GxE interaction effect from the residual variance. Instead, the GxE interaction effect is included in the residual error in the above model, and implicitly included in the heritability estimation of %HF.

Under the above linear mixed-effects model, heritability is defined as $\frac{\sigma_{g}^{2}}{\sigma_{g}^{2}+\sigma_{e}^{2}/J}$ (Holland et al., 2003), where *J* is the number of environments. To estimate heritability, we estimated all variance components ( $\sigma_{g}^{2},\;\sigma_{r}^{2},\sigma_{e}^{2}$ ) by the Restricted Maximum Likelihood (REML) approach using the R package lmer (Bates et al., 2015). Likelihood ratio tests were conducted, and p-values were computed accordingly. Heritability was estimated by plugging in the estimated variance components. The mean and standard error of mean %HF were computed by generalized least squares using the estimated variance-covariance matrix of the trait.

## 3 Results

### 3.1 Influence of *qshgd1* on haploid frailty

**Figure 1:**
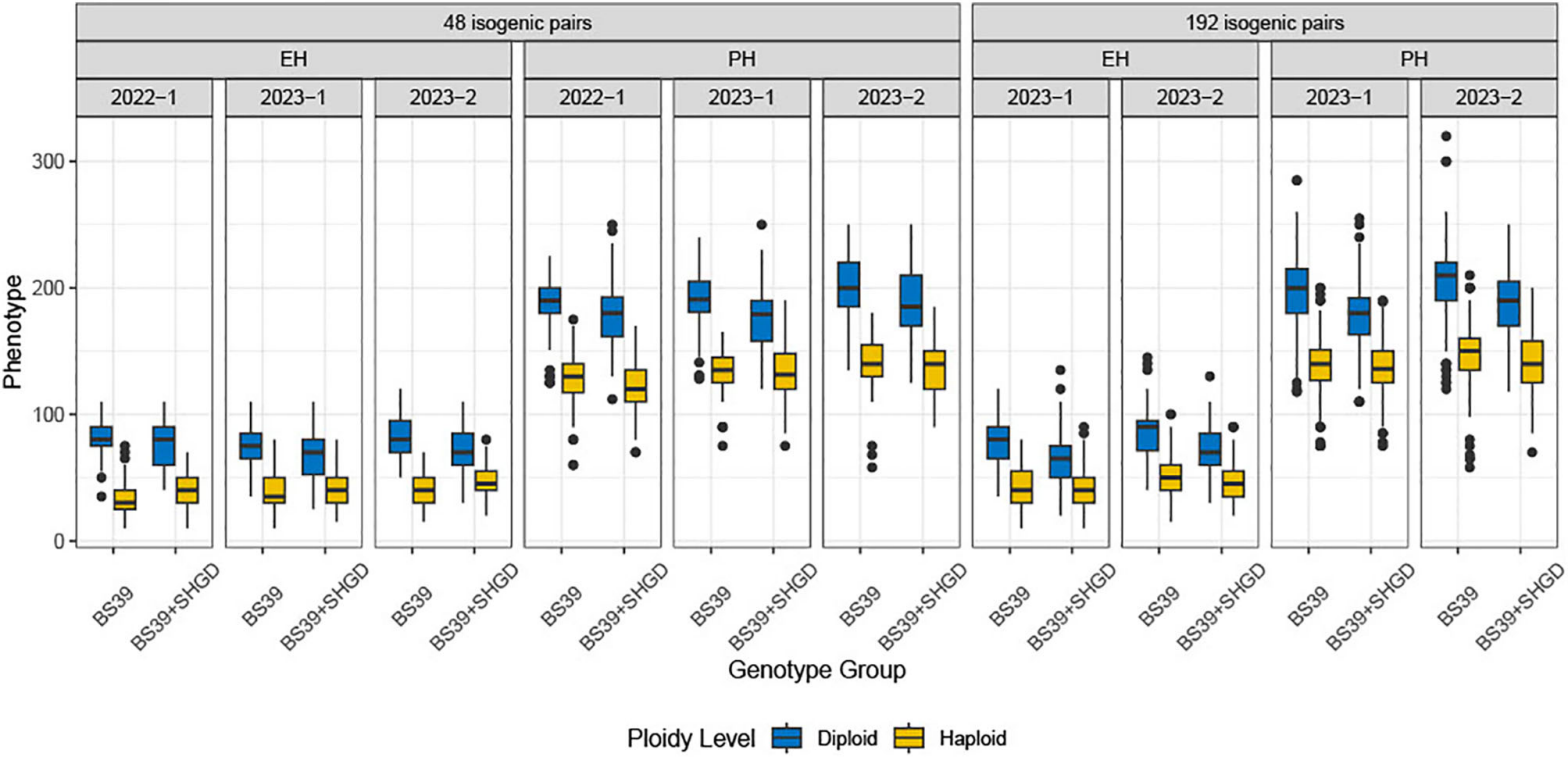
The distribution of observed PH, EH. Boxplots are plotted by genotype group, arranged by environment, traits, and datasets. Colors represent ploidy levels.

The variation in PH and EH traits between diploid and haploid genotype groups, as well as across different environments, clearly suggests that presence of *qshgd1* increases the performance of haploids. The mean HF% rates were significantly different between the BS39+SHGD group and the BS39 group, with the BS39+SHGD group showing a mean of 24% HF for PH and 36% HF for EH. In contrast, the BS39 group exhibited mean rates of 30% HF for PH and 44% for EH (**Figure 1**). The mean HF% rates were significantly lower in the BS39+SHGD group compared to the BS39 group for TL, SL and SD. BS39+SHGD group recorded 17% HF for TL, 18% HF for SL, and 11% HF for %SD. In contrast, the BS39 group showed mean rates of 25% HF for TL, 28% HF for SL, and 22% HF for %SD. The differences between the two groups were not statistically significant for FLL, FLW, and TB. (**Supplementary Figure 1**).

The higher temperatures and lower precipitation in July 2022 led to greater soil moisture depletion and drier conditions. In contrast, July 2023 experienced more favorable conditions, with higher precipitation, lower temperatures, and better soil moisture retention across all depths (**Supplementary Table 5**; **Supplementary Figure 3**).

**Figure 2:**
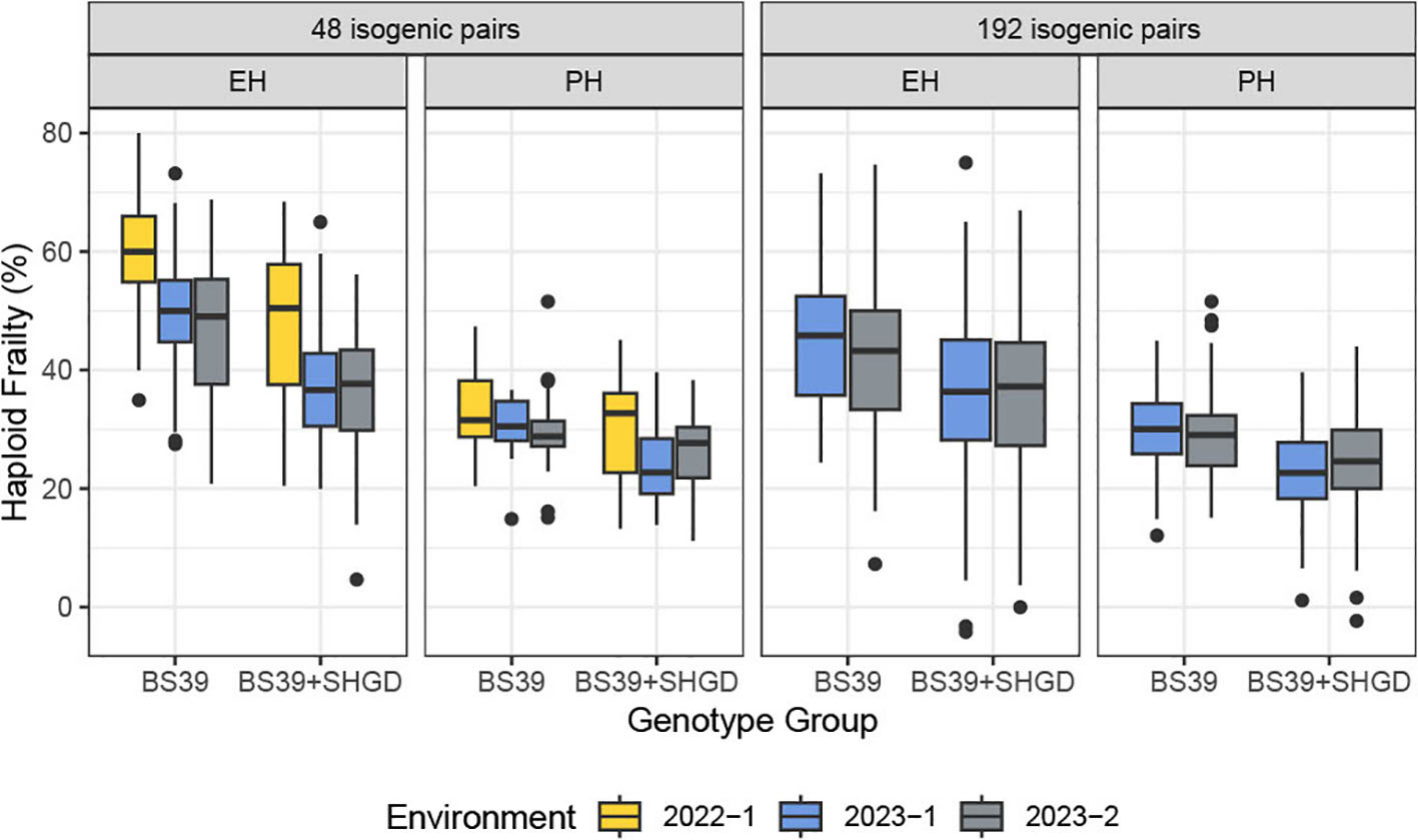
The distribution of computed %HF for PH, ear height EH. Boxplots are plotted by the genotype group, arranged by traits and datasets. Colors represent environments.

Analysis of the %HF data revealed a statistically significant difference between the years 2022 and the two staggered experiments in 2023 (2023-1 and 2023-2) for both the BS39+SHGD and BS39 isogenic pairs. Mean HF% values for PH (32%) and EH (54%) in 2022 were significantly higher compared to those observed in 2023-1 (27% for PH, 43% for EH) and 2023-2 (28% for PH, 41% for EH) (**Figure 2**). In contrast, the dataset from 2023-1and 2023-2 showed diminished environmental effects (**Supplementary Figure 2**). These findings suggest that while %HF was influenced by year-to-year differences, the effects of environmental factors were less pronounced in the two same year datasets in 2023.

### 3.2 Variation and heritability of HF% across morphological traits

**Figure 3:**
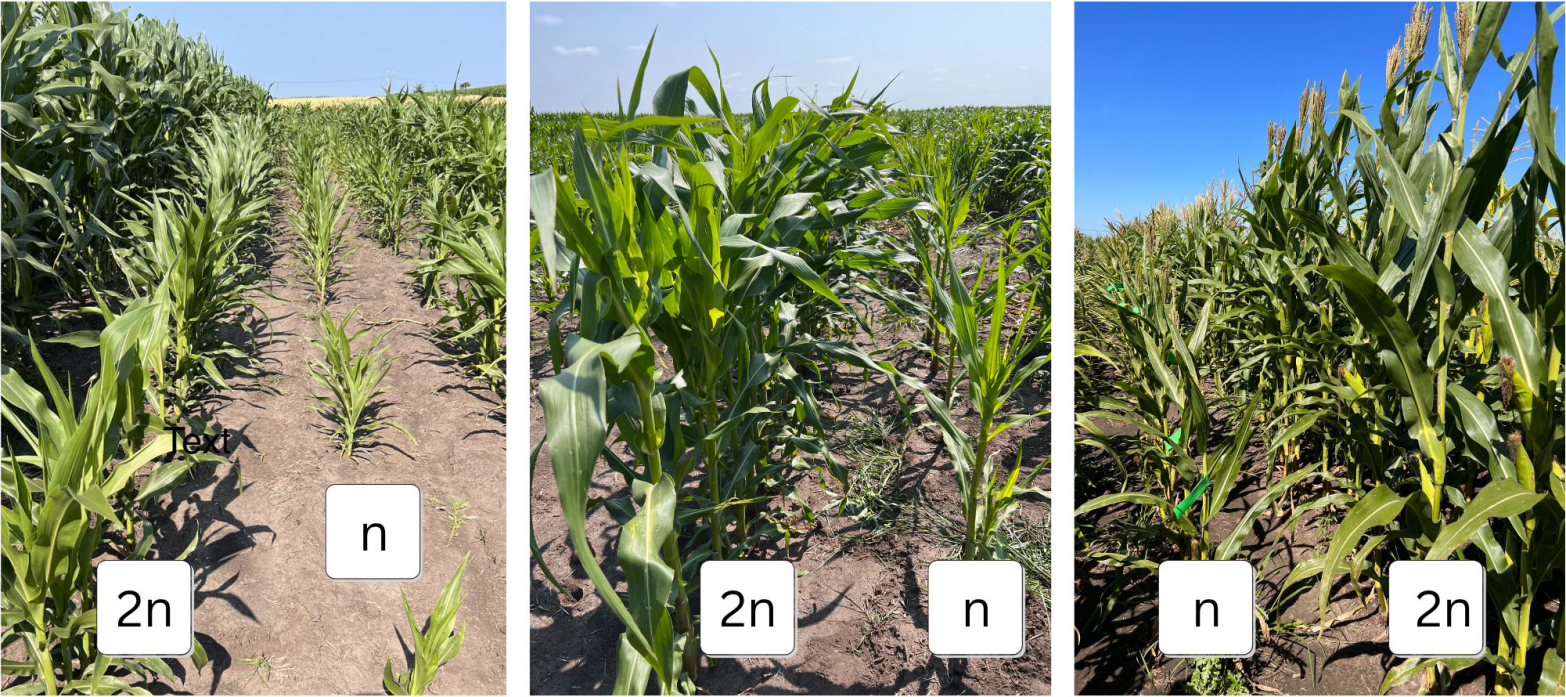
HIL-DH line pairs with pronounced differences between haploid (n) and diploid (2n) plants, e.g., for PH.

**Figure 4:**
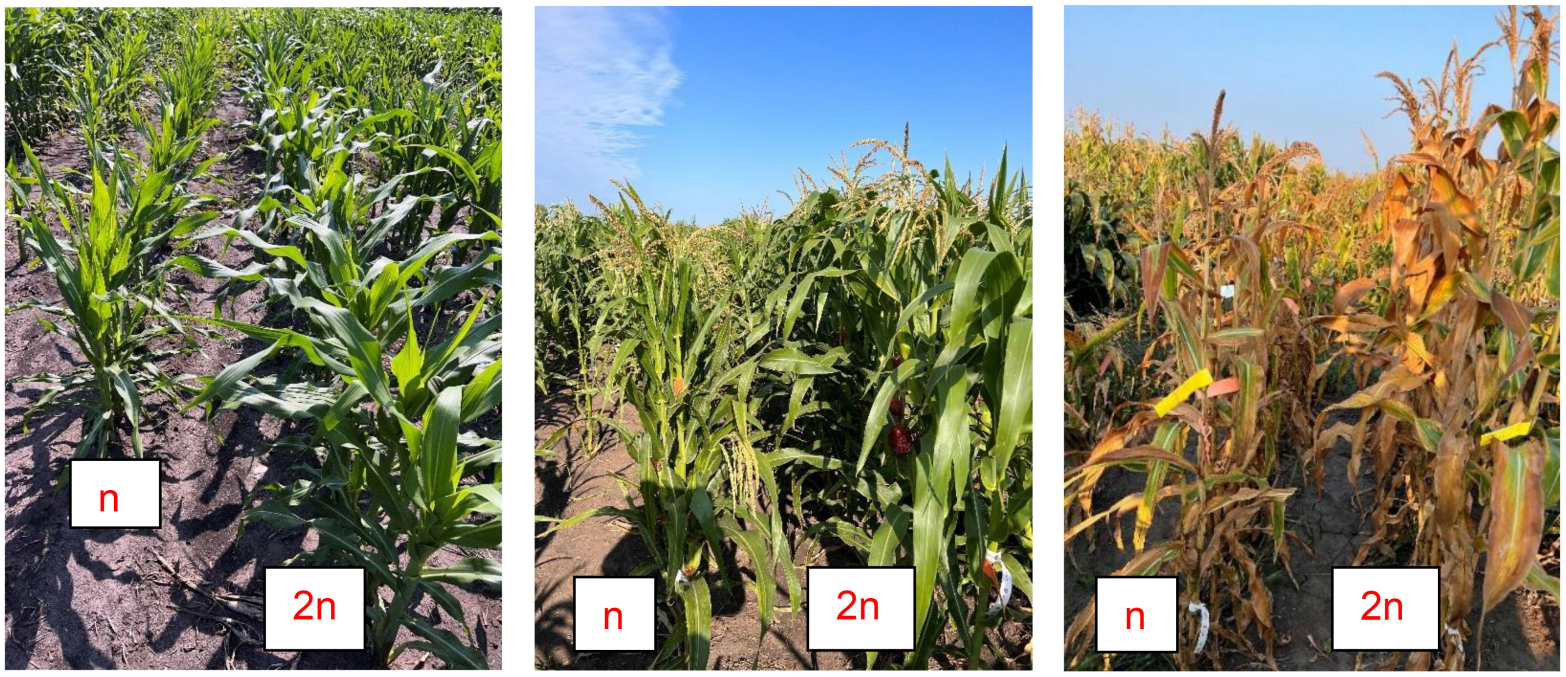
HIL-DH line pairs with small differences between haploid and diploid plants for PH.

HIL-DH line pairs exhibited a wide range of differences in PH between haploid and diploid plants, with some pairs showing large differences, reaching up to 59% HF (**Figure 3**), while others demonstrated minimal or no difference, with a minimum of 0% HF (**Figure 4**). Specifically, the BS39+SHGD group displayed narrow %HF ranges (minimum 0%, maximum 0.48%, mean 0.23%, and repeatability of 0.3 for 2023-1; minimum 0.01%, maximum 0.58%, mean 0.25%, and repeatability of 0.44 for 2023-2).

Genetic variation for %HF was significant and heritabilities of %HF for both PH and EH were moderate to high. Specifically, among 48 isogenic HIL – DH line pairs, the average %HF was higher (29% HF for PH) compared to a set of 192 isogenic genotypes (26% HF for PH). Heritability for %HF of PH was intermediate for the 48 isogenic pairs (heritability: 0.60). Both the average %HF and heritability values were higher among 48 isogenic genotypes (46% HF for EH; heritability: 0.72) compared to both PH (26% HF; 0.73) and EH values (39% HF; 0.65).

The average %HF value for FLW (16%) was closely aligned with that of FLL, with a lower heritability (0.15) observed among 192 HIL-DH lines pairs. TL %HF values (20%) closely approximated those of PH values. The heritability (0.48) was lower than for PH, but higher than for both FLL and FLW (**Table 1**; **Supplementary Table 1**).

**Table 1:** %HF and heritability for PH, EH, SL, TL, FLL, FLW, SD and TB.

Trait	Heritability	Est. HF	Genotype variance		Environmental variance	
			Estimate	P value	Estimate	P value
48 isogenic pairs						
PH	0.60	29% (25%, 33%)	0.00222	0	0.00058	0.001
EH	0.72	46% (36%, 55%)	0.01042	0	0.00455	0
192 isogenic pairs						
PH	0.74	26% (24%, 27%)	0.00358	0	0.00003	0.3317
EH	0.65	39% (36%, 41%)	0.00948	0	0.00008	0.4439
FLL	0.29	14% (12%, 16%)	0.00522	0.0211	0	1
FLW	0.15	16% (13%, 20%)	0.00252	0.2747	0.00025	0.414
TL	0.48	20% (18%, 21%)	0.00392	0	0	1
SL	0.60	21% (20%, 23%)	0.00651	0	0	1
SD	0.12	14% (3%, 25%)	0.01096	0.3847	0.00325	0.1384
TB	0.06	6% (3%, 8%)	0.00165	0.6786	0	1

The column Est. %HF reports the estimated mean %HF and its 95% confidence interval.

In the dataset comprising 48 isogenic pairs, the heritabilities for the BS39+SHGD group (PH: 0.70; EH: 0.70) were significantly higher than those of the BS39 group (PH: 0.30; EH: 0.56), with the difference being statistically highly significant (p=0.01). In the dataset of 192 isogenic pairs, the heritabilities for the BS39+SHGD group (PH: 0.69; EH: 0.69) were lower for PH compared to the BS39 group (PH: 0.74; EH: 0.59), while being higher for EH. The differences observed between the two groups were statistically significant (**Table 2**; p=0.05).

**Table 2:** Comparison of BS39 and BS39+SHGD groups for %HF and heritability for PH and EH.

Trait	BS39					BS39+SHGD				
	h^2^	Est. HF	Source	Variance	P value	h^2^	Est. HF	Source	Variance	P value
48 isogenic pairs										
PH	0.39	31% (28%, 34%)	Genotype	0.00105	0.0604	0.70	27% (21%, 32%)	Genotype	0.00286	0.0001
			Environment	0.00014	0.4454			Environment	0.00131	0.0004
EH	0.56	52% (42%, 61%)	Genotype	0.00541	0.0030	0.70	40% (30%, 49%)	Genotype	0.00846	0.0001
			Environment	0.00443	0.0002			Environment	0.00434	0.0002
192 isogenic pairs										
PH	0.74	30% (28%, 31%)	Genotype	0.00293	0.0000	0.68	24% (22%, 26%)	Genotype	0.00286	0.0000
			Environment	0.00000	1.0000			Environment	0.00011	0.0711
EH	0.59	44% (39%, 49%)	Genotype	0.00648	0.0005	0.64	36% (34%, 38%)	Genotype	0.00926	0.0000
			Environment	0.00049	0.1609			Environment	0.00000	1.0000

The column Est. %HF reports the estimated mean %HF and its 95% confidence interval.

The heritabilities for the BS39+SHGD group were lower for FLL (0.18) and SD (0.08) compared to the BS39 group, with values of 0.40 for FLL and 0.20 for SD. These differences were statistically significant (p=0.05). Conversely, the heritabilities for the BS39+SHGD group were significantly (p=0.05) higher for FLL (0.18) and SD (0.08) compared to the BS39 group. Additionally, heritabilities for the BS39+SHGD group for SL (0.48) and TB (0.06) did not show statistically significant differences when compared to the BS39 group (**Table 2**; **Supplementary Tables 2**-**4**).

Genetic variance components were significant for all traits (P=0.05), except for FLW, SL, and TB in the 192-line panel (**Table 1**). Moreover, genetic variance components were always larger than the environmental variance component for all traits and both panels.

### 3.3 Relationship of %HF with heritabilities across morphological traits in BS39 and BS39+SHGD isogenic line panels

**Figure 5:**
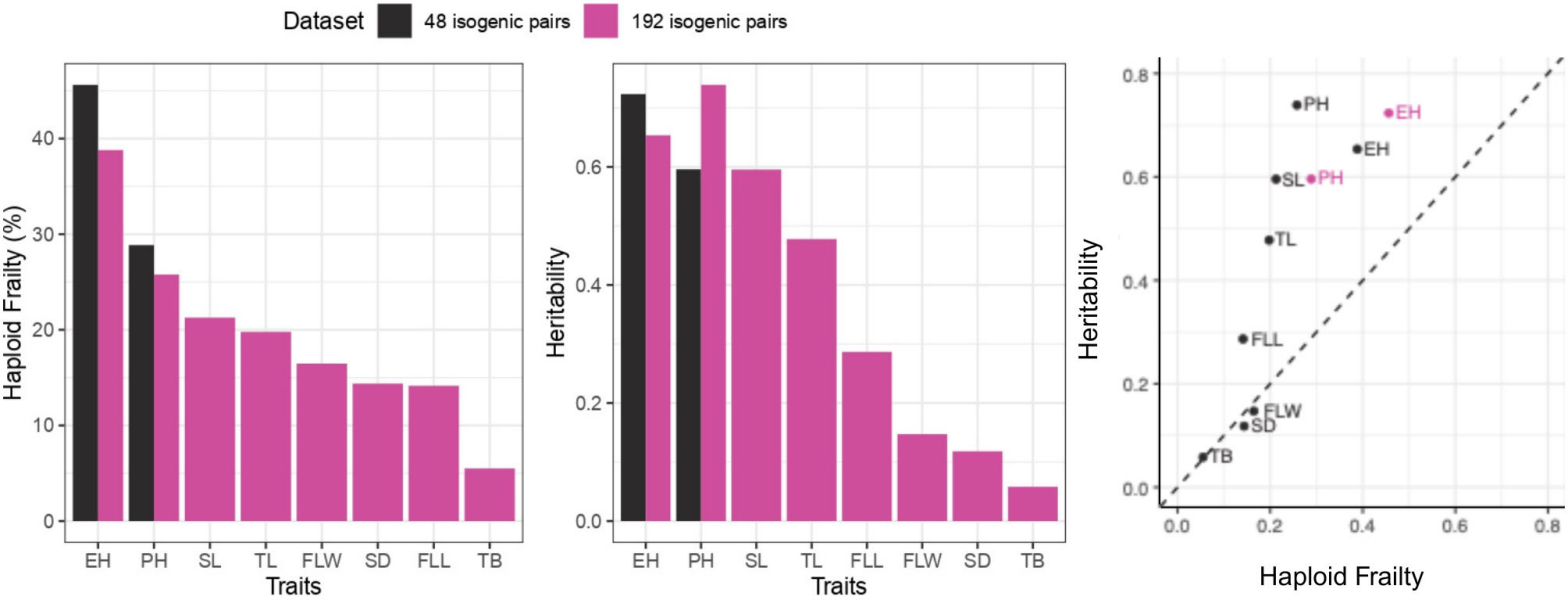
Summary by traits for %HF and heritability.

Heritabilities of traits such as PH, EH, SL, TL surpassed those of FLL, FLW, SD, TB traits. For the same traits (PH, EH, SL, TL) heritability values were also higher compared to other traits (**Figure 5**).

**Figure 6:**
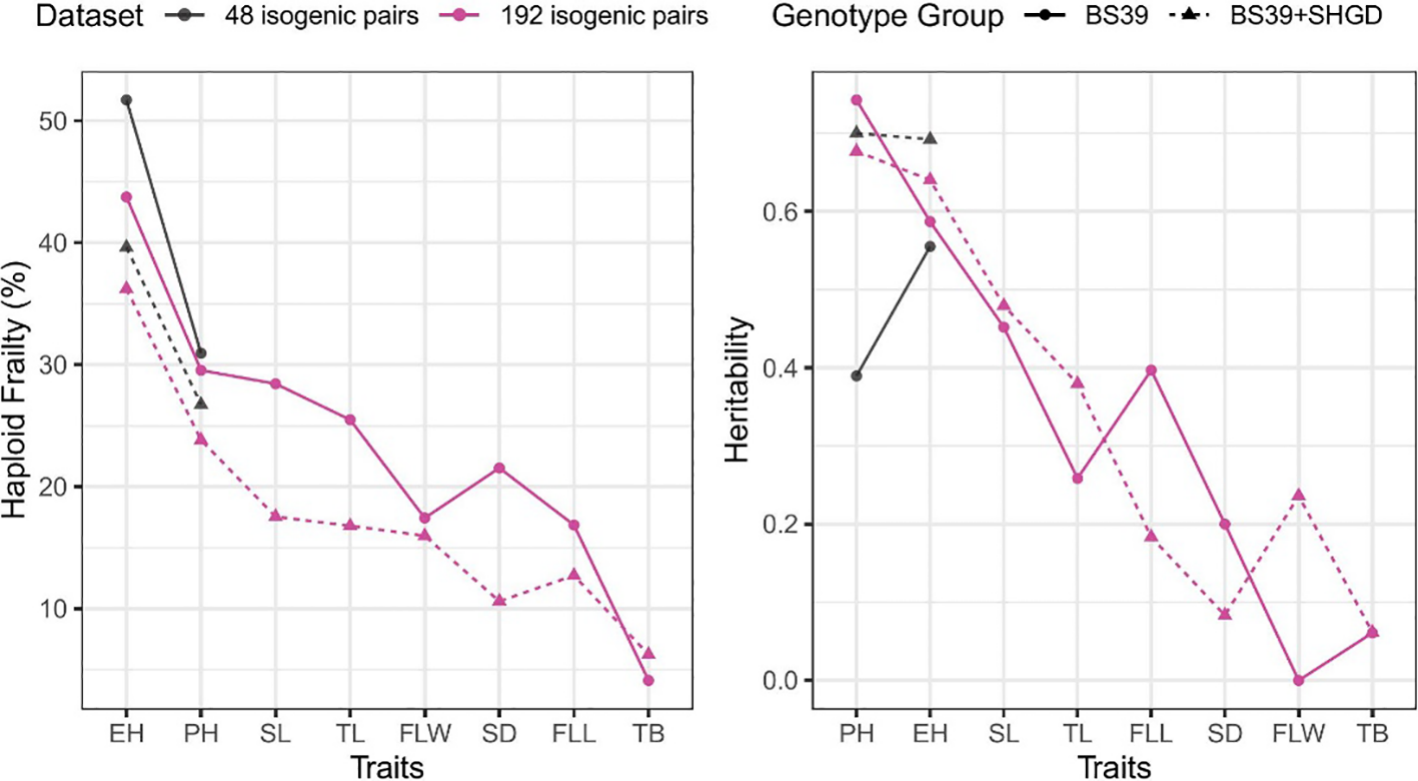
Summary by traits for mean %HF and heritability. Colors show different datasets. Line types and shapes show genotype groups.

Higher %HF and heritabilities values were found in BS39 compared to BS39+SHGD for PH. In case of EH, the heritability was lower for BS39 than for BS39+SHGD, although %HF was elevated. Values between BS39+SHGD and BS39 isogenic pairs differed, particularly for SL, TL, and SD. Notably, while the heritability for PH was elevated in BS39, BS39+SHGD had the higher heritability for EH. Additionally, PH exhibited the highest heritability values in both BS39 and BS39+SHGD genotypes. However, the overall trends with regard to levels of %HF and hertiabilities were similar between both isogenic line pair panels in BS39 and BS30+SHGD backgrounds (**Figure 6**).

## 4 Discussion

### 4.1 Data quality and experimental design

Heritability estimates for %HF for key traits such as PH and EH were intermediate to high (0.60–0.72). This is consistent with genetic variance being the largest variance component. While we aimed for a larger population of HILs with (228 DHs were induced), we did not produce enough haploid seed for 36 HILs. Nevertheless, 48 and later 192 HIL-DH isogenic line comparisons were sufficient to demonstrate that haploid frailty is heritable, that the extent of haploid frailty is trait-dependent, and that there is substantial variation for haploid frailty, which can be exploited by plant breeders.

### 4.2 Chase’s hypothesis

Chase (1964) reported an average haploid/diploid ratio of 0.72, corresponding to %HF of 28% for linear traits, which exceeded his expected value of 21%. He referred to this discrepancy as the “odd ploidy phenomenon.” In contrast, our study found an average %HF for linear traits that precisely matches Chase’s expected 21%, suggesting that the “odd ploidy phenomenon” does not apply to our dataset.

Our study, which was based on 192 HIL DH line pairs, revealed a range of %HF values across various morphological traits: 39% for EH, 26% for PH, 14% for both SD and FLL 16% for FLW, 20% for TL, and 21% for SL. These values are generally lower than those observed by Chase (1964): 30% for PH, 24% for SD, 23% for LL, and 28% for LW. This reduction in %HF values across traits may be due to the different genetic backgrounds used in both studies.

Additionally, the %HF range for plant height in our study was significantly wider, spanning from 0% to approximately 71%, compared to Chase’s range of 22% to 40%. This broader variability can be attributed to our larger sample size, which allowed us to capture a wider spectrum of %HF values, including outliers that may have been missed in Chase’s smaller sample of six line pairs.

Significant %HF variability was observed across all traits in our study, with values ranging from 3% to 55%. Certain traits, such as SD, FLL and FLW, exhibited particularly low %HF values of 3%, 12%, and 13%, respectively. This variability suggests that certain HIL-derived lines are phenotypically very similar to their isogenic DH counterparts. The heritable %HF variability among HILs is noteworthy, indicating potential for selection of genotypes with minimal %HF and thus similar attributes as isogenic diploids.

Chase hypothesized that traits considering plant parts would not show haploid frailty. Our findings support this hypothesis. Tassel branch number, the only plant part trait analyzed, showed the lowest %HF in our study (6%), lower than the 17% Chase reported. This alignment with Chase’s expectations suggests that the numbers of plant parts may indeed not differ substantially between haploid and diploid plants.

### 4.3 Environmental influences on %HF differences

In 2022, below-normal precipitation in July (2.90 inches, 1.72 inches below normal) led to substantial moisture stress. This stress likely negatively impacted haploid growth more than diploid growth. The %HF rates in 2022 increased to 32% for PH and 54% for EH. With their smaller root structures, haploids struggled to access sufficient water resources, resulting in average haploid heights for PH of 124.3 cm compared to 181.3 cm for diploids. This aligns with Chase’s (1964) observation that haploid plants, due to their smaller root systems, face greater difficulty in growth under limited water conditions.

In contrast, the cooler-than-normal average temperatures in July of 2023 (71.8°F, 1.65°F below normal for both months) likely reduced evaporation rates, mitigating moisture stress and thus easing the environmental stress on haploids. Consequently, haploid frailty rates in 2023 were lower than in 2022, with %HF rates for PH falling to 27% in the 2023-1 dataset and 28% in the 2023-2 dataset, while for EH, %HF rates dropping to 43% and 41%, respectively. For PH, the average haploid height was 133.5 cm in the 2023-1 dataset and 138.7 cm in the 2023-2 dataset, with corresponding diploid heights of 184 cm and 193 cm, respectively.

This annual variation in %HF illustrates the increased vulnerability of haploids under the harsher conditions of 2022, highlighting that haploid plants are more susceptible to environmental stress. In contrast, the stable climate in 2023 appears to have contributed to more consistent, milder vulnerability rates, with less pronounced environmental variability in %HF between the 2023-1 and 2023-2 datasets. This suggests that a relatively stable growing environment, such as the one experienced in 2023, can help mitigate haploid vulnerability, emphasizing the importance of environmental stability for reducing stress impacts on haploids.

Haploid-diploid ratio have been associated to ecological dissimilarities between phases in fecundity, recruitment, growth or mortality (Engel et al., 2001; Thornber and Gaines, 2004; Vieira et al., 2018). Similarly, the variations in %HF in maize suggests the potential for overcoming evolutionary constraints, much like how mosses maintain a dominant haploid phase.

### 4.4 Impact of SHGD on %HF

The impact of SHGD on %HF was substantial, offering solutions to some of the inherent challenges of haploidy, including reduced vigor and sterility. Specifically, the presence of the *qshgd1* locus in the BS39+SHGD group significantly reduced %HF across various traits, except for tassel branch number, when compared to the BS39 genotype.

Our results indicate that maize genotypes carrying the SHGD trait and qshgd1 locus exhibit improved haploid vigor and fertility, likely due to early haploid genome doubling. If validated across multiple genetic backgrounds, qshgd1 could be leveraged in breeding programs to enhance DH production efficiency, reducing the reliance on artificial doubling methods. Further research is required to establish its efficacy in diverse maize populations and to optimize breeding strategies for its effective deployment.

### 4.5 Implications for plant breeding

The implications of our study of %HF for plant breeding are profound, as the use of DH lines offers many advantages for accelerating breeding programs in major crops like maize, wheat, and barley. However, the limitations posed by %HF, such as reduced vigor and sterility in haploid plants, pose challenges that must be overcome to maximize the utility of DH lines. The identification of genetic variability for %HF, as seen in traits like PH and EH, presents opportunities for breeders to selectively target and reduce haploid frailty. The integration of SHGD is particularly promising, as it can alleviate some of the sterility issues and lead to the production of more vigorous haploid lines.

The potential of SHGD to improve the fertility and vigor of haploid plants opens up new possibilities for plant breeders. Breeding programs can utilize SHGD to create more fertile and resilient haploid plants, increasing the chance of developing DH lines, which are crucial for advancing the genetic gains in crop improvement.

## Data Availability

The original contributions presented in the study are included in the article/**Supplementary Material**. Further inquiries can be directed to the corresponding author.

## Supplementary material

Supplementary Figure 1 The distribution of additional six traits collected in 2023. Boxplots are plotted by the genotype group, faceted by environment and traits. Colors represent ploidy levels.

Supplementary Figure 2 The distribution of haploid frailty for additional six traits collected in 2023. Boxplots are plotted by the genotype group, faceted by datasets and traits. Colors represent environments.

Supplementary Figure 3 Volumetric soil water content (%), soil temperature °F and temperature °F for 2022-2023 (Iowa Environmental Mesonet of Iowa State University).

Supplementary Table 1 Variance components for PH, EH, FLL, FLW, TL, SL, SD and TB.

Supplementary Table 2 Comparison of BS39 and BS39+SHGD groups on mean HF and heritability for FLL, FLW, TL, SL, SD and TB. The column Est. %HF reports the estimated mean %HF and its 95% confidence interval.

Supplementary Table 3 Variance components from BS39 group for PH, EH, FLL, FLW, TL, SL, SD and TB.

Supplementary Table 4 Variance components from BS39+SHGD group for PH, EH, FLL, FLW, TL, SL, SD and TB.

Supplementary Table 5 Average daily temperature and precipitation data for May to September (2022-2023). Mean*: Average of the years 1973-2024. (Iowa Environmental Mesonet of Iowa State University).
