# Supplementary material for: Genetic variation and heritability of haploid frailty in maize
Source: Front Plant Sci. 2025 Jun 3;16:1572901. doi: 10.3389/fpls.2025.1572901 (PMC12188443; doi:10.3389/fpls.2025.1572901)
Supplement: Supplementary Figure 1 — The distribution of additional six traits collected in 2023. Boxplots are plotted by the genotype group, faceted by environment and traits. Colors represent ploidy levels. [file DataSheet1.pdf]

Supplemental Figures and Tables

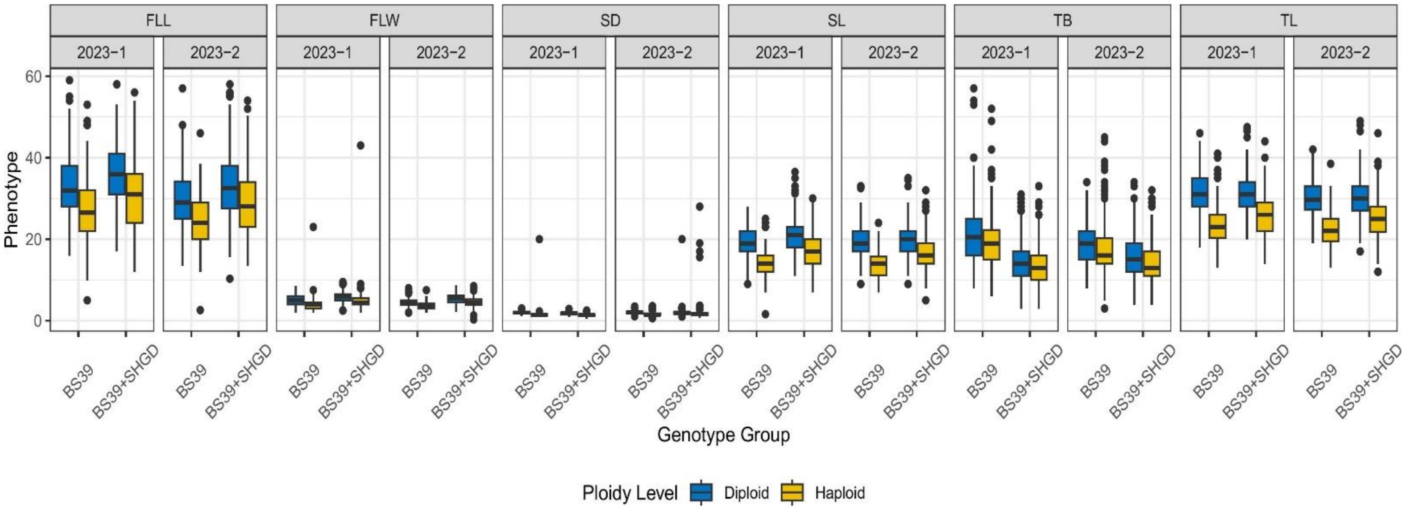

Supplemental Figure 1. The distribution of additional six traits collected in 2023. Boxplots are plotted by the genotype group, faceted by environment and traits. Colors represent ploidy levels.

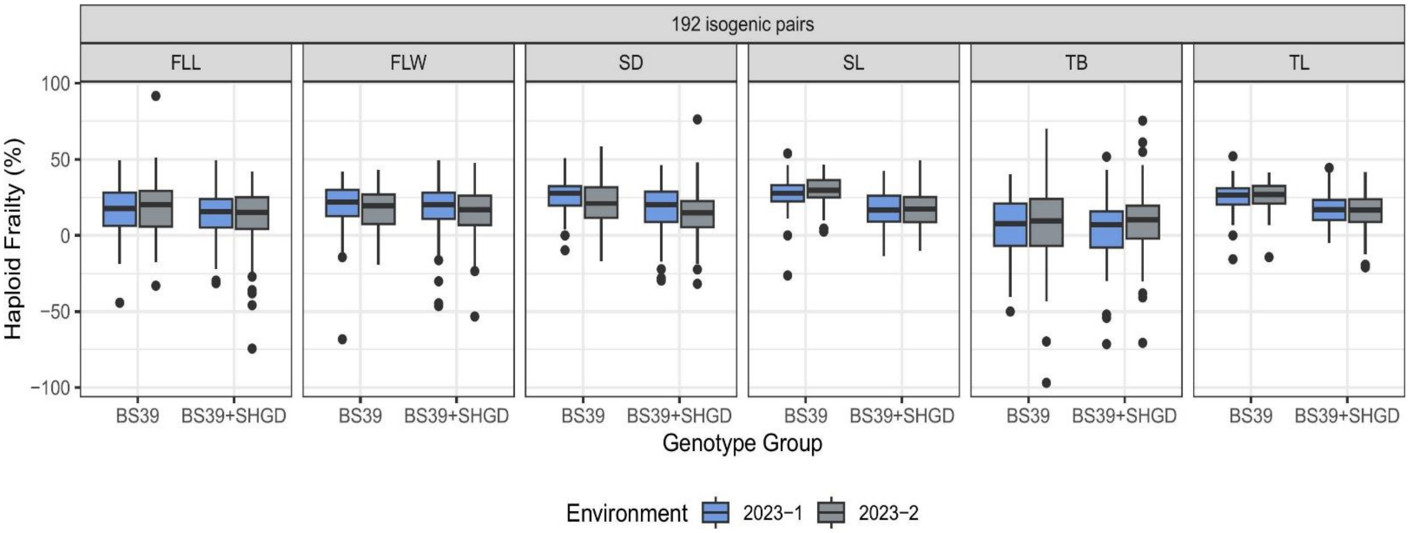

Supplemental Figure 2. The distribution of haploid frailty for additional six traits collected in 2023. Boxplots are plotted by the genotype group, faceted by datasets and traits. Colors represent environments.

Supplemental Table 1: Variance Components for PH, EH, FLL, FLW, TL, SL, SD and TB.

	Genotype Variance				Environment Variance			
Trait	Estimate	2.5 %	97.5 %	p value	Estimate	2.5 %	97.5 %	p value
48 isogenic pairs								
PH	0.00222	0.0011	0.0040	0.0000	0.00058	0.0001	0.0041	0.0010
EH	0.01042	0.0060	0.0178	0.0000	0.00455	0.0007	0.0291	0.0000
192 isogenic pairs								
PH	0.00358	0.0027	0.0047	0.0000	0.00003	0.0000	0.0006	0.3317
EH	0.00948	0.0066	0.0129	0.0000	0.00008	0.0000	0.0017	0.4439
FLL	0.00522	0.0007	0.0099	0.0211	0.00000	0.0000	0.0011	1.0000
FLW	0.00252	0.0000	0.0072	0.2747	0.00025	0.0000	0.0036	0.4140
TL	0.00392	0.0021	0.0059	0.0000	0.00000	0.0000	0.0005	1.0000
SL	0.00651	0.0043	0.0091	0.0000	0.00000	0.0000	0.0007	1.0000
SD	0.01096	0.0000	0.0364	0.3847	0.00325	0.0000	0.0360	0.1384
TB	0.00165	0.0000	0.0080	0.6786	0.00000	0.0000	0.0021	1.0000

Supplemental Table 2: Comparison of BS39 and BS39+SHGD groups on mean HF and heritability for FLL, FLW,

TL, SL, SD and TB. The column Est. %HF reports the estimated mean %HF and its 95% confidence interval.

	BS39					BS39+SHGD				
Trait	Heritability	Est. HF	Source	Variance	p-value	Heritability	Est. HF	Source	Variance	p-value
FLL	0.40	17% (13%, 21%)	Genotype	0.00859	0.0416	0.18	13% (11%, 15%)	Genotype	0.00292	0.2657
			Environment	0.00000	1.0000			Environment	0.00002	0.9408
FLW	0.00	17% (15%, 20%)	Genotype	0.00000	1.0000	0.24	16% (13%, 19%)	Genotype	0.00473	0.1368
			Environment	0.00023	0.7238			Environment	0.00007	0.8584
TL	0.26	25% (23%, 28%)	Genotype	0.00150	0.2277	0.38	17% (15%, 19%)	Genotype	0.00261	0.0084
			Environment	0.00000	1.0000			Environment	0.00005	0.6574
SL	0.45	28% (25%, 32%)	Genotype	0.00331	0.0161	0.48	18% (16%, 19%)	Genotype	0.00421	0.0003
			Environment	0.00026	0.3285			Environment	0.00000	1.0000
SD	0.20	22% (16%, 27%)	Genotype	0.00893	0.3669	0.08	11% (-6%, 27%)	Genotype	0.00952	0.6271
			Environment	0.00000	1.0000			Environment	0.00728	0.1063
TB	0.06	4% (-1%, 9%)	Genotype	0.00227	0.8001	0.06	6% (4%, 9%)	Genotype	0.00146	0.7223
			Environment	0.00000	1.0000			Environment	0.00006	0.9147

Supplemental Table 3: Variance Components from BS39 group for PH, EH, FLL, FLW, TL, SL, SD and TB.

	Genotype Variance				Environment Variance			
Trait	Variance	2.5 %	97.5 %	p-value	Variance	2.5 %	97.5 %	p-value
**48 isogenic pairs**								
PH	0.00105	0.0000	0.0029	0.0604	0.00014	0.0000	0.0018	0.4454
EH	0.00541	0.0015	0.0128	0.0030	0.00443	0.0006	0.0294	0.0002
**192 isogenic pairs**								
PH	0.00293	0.0017	0.0046	0.0000	0.00000	0.0000	0.0004	1.0000
EH	0.00648	0.0027	0.0113	0.0005	0.00049	0.0000	0.0068	0.1609
FLL	0.00859	0.0000	0.0183	0.0416	0.00000	0.0000	0.0034	1.0000
FLW	0.00000	0.0000	0.0046	1.0000	0.00023	0.0000	0.0051	0.7238
TL	0.00150	0.0000	0.0042	0.2277	0.00000	0.0000	0.0010	1.0000
SL	0.00331	0.0005	0.0066	0.0161	0.00026	0.0000	0.0040	0.3285
SD	0.00893	0.0000	0.0299	0.3669	0.00000	0.0000	0.0072	1.0000
TB	0.00227	0.0000	0.0204	0.8001	0.00000	0.0000	0.0065	1.0000

Supplemental Table 4: Variance Components from BS39+SHGD group for PH, EH, FLL, FLW, TL, SL, SD and TB.

	Genotype Variance				Environment Variance			
Trait	Variance	2.5 %	97.5 %	p-value	Variance	2.5 %	97.5 %	p-value
**48 isogenic pairs**								
PH	0.00286	0.0012	0.0061	0.0001	0.00131	0.0002	0.0091	0.0004
EH	0.00846	0.0034	0.0184	0.0001	0.00434	0.0006	0.0296	0.0002
**192 isogenic pairs**								
PH	0.00286	0.0019	0.0041	0.0000	0.00011	0.0000	0.0015	0.0711
EH	0.00926	0.0058	0.0135	0.0000	0.00000	0.0000	0.0009	1.0000
FLL	0.00292	0.0000	0.0075	0.2657	0.00002	0.0000	0.0024	0.9408
FLW	0.00473	0.0000	0.0113	0.1368	0.00007	0.0000	0.0032	0.8584
TL	0.00261	0.0006	0.0048	0.0084	0.00005	0.0000	0.0013	0.6574
SL	0.00421	0.0019	0.0069	0.0003	0.00000	0.0000	0.0006	1.0000
SD	0.00952	0.0000	0.0493	0.6271	0.00728	0.0000	0.0780	0.1063
TB	0.00146	0.0000	0.0082	0.7223	0.00006	0.0000	0.0039	0.9147

Supplemental Table 5. Average Daily Temperature and Precipitation Data for May to September (2022-2023). Mean*: Average of the years 1973-2024.(Iowa Environmental Mesonet of Iowa State University).

	Year	May	June	July	August	September
Average Daily High Temperature [F]	2022	72.12	83.66	84.32	82.46	76.22
	2023	75.95	82.28	81.12	82.75	79.64
	Mean*	71.01	80.70	84.20	82.20	76.11
Average Daily Low Temperature [F]	2022	51.86	62.72	65.74	63.01	54.20
	2023	52.61	61.34	62.48	63.70	56.48
	Mean*	48.51	58.6	62.70	60.79	51.70
Average Daily Temperature (high+low)/2 [F]	2022	61.99	73.19	75.03	72.73	65.21
	2023	64.28	71.81	71.80	73.23	68.06
	Mean*	59.76	69.65	73.45	71.50	63.90
Precipitation Totals [inch]	2022	3.97	7.36	2.90	2.60	2.01
	2023	2.98	4.81	4.62	2.24	0.28
	Mean*	5.17	5.38	4.80	4.92	3.32

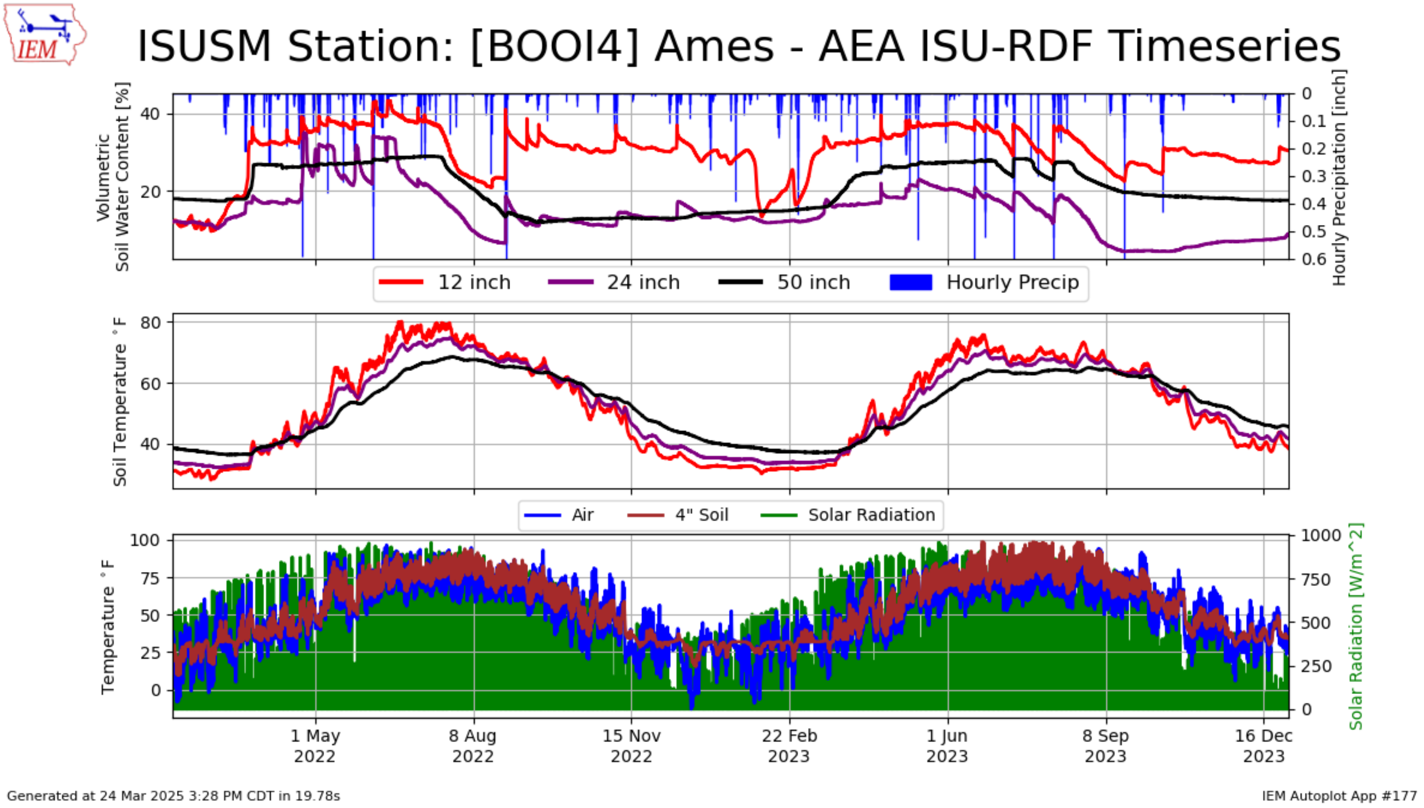

Supplemental Figure 3. Volumetric Soil Water Content (%), Soil Temperature °F and Temperature °F for 2022-2023 (Iowa Environmental Mesonet of Iowa State University).
